# Internet Use, Depression, and Anxiety in a Healthy Adolescent Population: Prospective Cohort Study

**DOI:** 10.2196/mental.8471

**Published:** 2018-05-22

**Authors:** Robyn Pauline Thom, David S Bickham, Michael Rich

**Affiliations:** ^1^ Harvard Longwood Psychiatry Residency Training Program Brigham and Women's Hospital & Beth Israel Deaconess Medical Center Boston, MA United States; ^2^ Center on Media and Child Health Boston Children's Hospital Boston, MA United States

**Keywords:** mental health, psychiatric disorders, internet use, social networking sites

## Abstract

**Background:**

Psychiatric disorders, including conduct disturbances, substance abuse, and affective disorders, emerge in approximately 20% of adolescents. In parallel with the rise in internet use, the prevalence of depression among adolescents has increased. It remains unclear whether and how internet use impacts mental health in adolescents.

**Objective:**

We assess the association between patterns of internet use and two mental health outcomes (depression and anxiety) in a healthy adolescent population.

**Methods:**

A total of 126 adolescents between the ages of 12 and 15 years were recruited. Participants reported their typical computer and internet usage patterns. At baseline and one-year follow-up, they completed the Beck Depression Index for primary care (BDI-PC) and the Beck Anxiety Inventory for Primary Care (BAI-PC). Individual linear regressions were completed to determine the association between markers of internet use at baseline and mental health outcomes at one-year follow-up. All models controlled for age, gender, and ethnicity.

**Results:**

There was an inverse correlation between minutes spent on a favorite website per visit and BAI-PC score. No association was found between internet use and BDI-PC score.

**Conclusions:**

There is no relationship between internet use patterns and depression in adolescents, whereas internet use may mitigate anxiety in adolescents with higher levels of baseline anxiety.

## Introduction

### Background

Psychiatric disorders, including conduct disturbances, substance abuse, and affective disorders, emerge in approximately 20% of adolescents [1]. Internet use is pervasive among teens, with 92% of American teens reporting daily internet use and 24% reporting nearly constant use [2]. The developmental tasks of adolescence include establishing autonomy, developing a self-image, and creating healthy social connections [1]. It remains unclear how or whether internet use affects these developmental tasks. In parallel with the rise in internet use, prevalence of depression among adolescents has increased from 8.7% in 2005 to 11.3% in 2014 [3]. This observation of a parallel rise in internet use and depression has raised questions, spurred research, and fueled controversy.

Existing research on the association between internet use and mental health outcomes has focused on the effect of social networking site (SNS) use in the young adult and college population. As reviewed by Primack et al, there is a great deal of controversy as to whether overall SNS use is associated with mental health outcomes in transitional age youth [4]. While some studies suggest that SNS users experience positive mental health effects such as increased perceived peer support, social capital, and overall life satisfaction [5,6], others show a correlation between increased SNS use and depression [7,8]. Less research has examined the effect of general internet use and internet use patterns on middle and high school-aged youth. Interestingly, one study found that both low-intensity and high-intensity self-reported internet use was associated with poorer mental health outcomes in adolescents [9]. Whether specific internet use patterns, such as time spent on interactive versus noninteractive sites and weekday versus weekend use, are associated with mental health outcomes remains unknown. Our prospective cohort study examines associations between self-reported patterns of internet use and depression and anxiety in a community-based sample of adolescents.

## Methods

### Study Participants

In 2009, English-speaking participants between the ages of 12 and 15 years were recruited from public schools, after-school programs, and community programs in a small New England city (Table 1). Participants provided assent and parents provided written consent for study participation. Research ethics approval was granted by the Committee on Clinical Investigation at Boston Children’s Hospital. Data were collected using the Measuring Youth Media Exposure procedure for recall estimation [10]. Media use and mental health status were measured annually with a computer-assisted self-interview in which participants reported typical amounts of time using computers and internet, favorite websites, how often and for how long they visited them each time. Favorite websites were assessed as interactive or not interactive, where any website that primarily functioned to connect people was coded as interactive. Examples of interactive sites included SNS (eg, Facebook), email sites (eg, Gmail), and online chat sites. The favorite website variable was subsequently treated as a dichotomous variable (interactive versus noninteractive) for statistical analysis.

### Data Collection

At baseline and one-year follow-up, participants completed the Beck Depression Inventory for Primary Care (BDI-PC) and the Beck Anxiety Inventory for Primary Care (BAI-PC), validated screening tools for detecting anxiety and mood disorders in adolescents [11]. 

**Table 1:** Participant characteristics (USA 2009).

Characteristics		Value
**Total participants, n**		
	At start	126
	At 1-year follow-up	103
Female gender, n (%)		59 (46.9)
Age, mean (range)		14.04 (12.56-15.94)
Nonwhite race, n (%)		57 (45.2)
**Internet use, mean (SD)**		
	School day in min	53.37 (59.62)
	Weekend in min	91.92 (128.25)
**Beck Depression Inventory for Primary Care, mean (SD)**		
	Baseline	2 (6.2)
	Follow-up	1.8 (2.5)
**Beck Anxiety Inventory for Primary Care, mean (SD)**		
	Baseline	12 (2.9)
	Follow-up	11.2 (6.1)
**Ownership, n (%)**		
	Mobile internet device	61 (48.4)
	Personal laptop	37 (29.4)
	Personal cell phone	86 (68.3)
**Parental education (highest), %**		
	Less than high school	17.4
	Completed high school	30.6
	At least some college	52

For each of these screening tools, participants ranked seven symptoms on a scale of 0 to 3, where 0 indicated the symptom was not present and 3 indicated a high severity of the symptom. Examples of questions about depression included sadness, pessimism, and loss of interest. Examples of questions assessing anxiety included worrying at bedtime, worrying about parents, and feelings of nervousness. Each of the scales is scored from 0 to 21, where scores of 0-3 indicate minimal, 4-6 mild, 7-9 moderate, and 10-21 severe symptom burden.

Data were analyzed using Statistical Package for the Social Sciences (SPSS) v.20 (IBM) software. Statistical significance was defined as *P* value <.05. Individual linear regressions were completed to determine the association between internet use at baseline and mental health outcomes at one-year follow-up. All models controlled for age, gender, race, and baseline depression and anxiety.

## Results

A total of 126 adolescents were included at baseline. Of these, 23 participants were lost to follow-up and excluded from the analysis. Demographic as well as mean baseline and follow-up BDI-PC and BAI-PC are summarized in Table 1. A total of 48.4% of participants owned a mobile internet device, such as a cell phone with internet capabilities.

Linear regression beta coefficients between internet use and mental health outcomes are shown in Tables 2 and 3. There was an inverse correlation between minutes spent on a favorite website per visit and anxiety (BAI-PC score). No association was found between internet use and depression (BDI-PC score).

**Table 2:** Linear regression analysis using internet use to predict depression scores at 1-year follow-up.

Internet use variable^a^	Unstandardized coefficients		Standardized coefficient (beta)		*t*		*P* value	
	B	SE^b^						
Favorite site interactive?	.62	.71		.09		.88		.38
Visits/week on favorite site	.43	.29		.16		1.51		.14
Visits/week on interactive site	.21	.18		.12		1.20		.23
Minutes on favorite site/visit	–.29	.31		–.10		–.94		.35
Minutes on computer/school day	.00	.01		.01		.12		.91
Minutes on internet/school day	.00	.01		.06		.48		.63
Minutes email or chat/school day	.00	.01		–.06		–.51		.61
Minutes on computer/weekend day	.00	.00		.02		.19		.85
Minutes on internet/weekend day	.00	.00		.06		.49		.63
Minutes email or chat/weekend day	.00	.00		.05		.40		.69

^a^Covariates included age, gender, race, and baseline depression.

^b^SE: standard error.

**Table 3:** Linear regression analysis using internet use to predict anxiety scores at 1-year follow-up.

Internet use variable^a^	Unstandardized coefficients		Standardized coefficient (beta)	*t*	*P* value
	B	SE^b^			
Favorite site interactive?	–.01	1.05	.00	–.01	.99
Visits/week on favorite site	.11	.43	.03	.27	.79
Visits/week on interactive site	–.20	.26	–.08	–.76	.45
Minutes on favorite site/visit	–1.06	.44	–.24	–2.39	.02
Minutes on computer/school day	–.01	.01	–.12	–.93	.35
Minutes on internet/school day	–.01	.01	–.09	–.74	.46
Minutes email or chat/school day	.00	.01	.02	.19	.85
Minutes on computer/weekend day	.00	.00	.01	.09	.93
Minutes on internet/weekend day	–.01	.00	–.20	–1.79	.08
Minutes email or chat/weekend day	.00	.00	–.09	–.77	.44

^a^Covariates included age, gender, race, and baseline depression.

^b^SE: standard error.

## Discussion

### Principal Findings

We found no relationship between internet use and depression in healthy adolescents. While time in minutes spent on each visit to a favorite website was negatively correlated with anxiety scores, no other measures of internet use were correlated with anxiety scores. Particularly, there was no relationship between preferred use of interactive websites and anxiety, suggesting that use of interactive websites is not associated with worse mental health outcomes. The correlation between increased time spent on a favorite website per visit and lower anxiety scores may indicate that adolescents are using their favorite website (either interactive or noninteractive) as a self-soothing tool to reduce anxiety. Overall, our results suggest a minimal effect of internet use on depression and anxiety.

The literature regarding the effect of internet use on mental health outcomes remains controversial [4]. Many of the studies that demonstrate a correlation between internet use and poor mental health outcomes have focused on problematic internet use, a pattern of internet use with features resembling addiction, such impulsive or risky use resulting in social, occupational, or emotional impairment [12,13]. A prospective study of adolescents demonstrated that pathologic internet use at baseline was 1.5 times more highly associated with depression at the 9-month follow-up, compared with nonpathologic use [13]. The studies that have demonstrated no association between internet use and mental health outcomes have typically focused more on moderate, or nonpathologic use. One study of adolescent university students showed no relationship between SNS use and depression [14]. Additionally, Belanger et al demonstrated a U-shaped relationship between self-reported intensity (duration) of internet use and depression. Adolescents with high and low internet use had higher depressive scores, compared with moderate users [9]. The results of our study parallel this work, as the participants of this study had a mean internet use of less than one hour on school days and approximately 1.5 hours on weekends, representing moderate internet use.

Importantly, the generalizability of our findings in the present is limited by the fact that the data were collected in 2009. Internet use has become much more pervasive over the last several years, particularly with the increased affordability and accessibility of handheld devices including smart-phones and tablets. The landscape and functions offered by SNS have also evolved, now with increased advertising, instant messaging, and media uploading capabilities. Our data may not capture how the increased role of the internet in forming and sustaining relationships affects mental health. Generalization of our results is also limited by the small sample size and reliance on self-reported internet use measures which may be limited in reliability. Further research that includes methodologies that allow for more contextualized measures of internet use, such as passive versus active use, social setting, and motivations for use, may provide additional information relevant to how patterns of internet use affect mental health in adolescents. Another limitation was that while our model controlled for age, gender, race, and baseline BDI-PC and BAI-PC scores, we were unable to control for other risk factors for depression and anxiety such as family history of mental illness, coping style, and trauma history. Finally, the population studied was a community-based population, and thus, these results may not be generalizable to a clinic-based population, such as those already receiving treatment for depression, anxiety, or other mental illnesses. Given these limitations and inconsistencies in previous studies, further research must be done.

### Conclusion

We were unable to detect an association between internet use and depression and anxiety in a community sample of adolescents.

## Abbreviations

BAI-PC: Beck Anxiety Inventory for Primary Care
BDI-PC: Beck Depression Inventory for Primary Care
SNS: social networking site
SPSS: Statistical Package for the Social Sciences
